# Preoperative localisation of nonpalpable breast lesions using magnetic markers in a tertiary cancer centre

**DOI:** 10.1186/s41747-022-00280-2

**Published:** 2022-07-06

**Authors:** Antonella Petrillo, Raimondo Di Giacomo, Emanuela Esposito, Paolo Vallone, Sergio Venanzio Setola, Mauro Mattace Raso, Vincenza Granata, Maria Luisa Barretta, Claudio Siani, Chiara Rinaldo, Ivana Donzelli, Ugo Marone, Maria Teresa Melucci, Alfredo Fucito, Ruggero Saponara, Maurizio Di Bonito, Roberta Fusco, Massimo Rinaldo, Franca Avino

**Affiliations:** 1 Radiology Division, “Istituto Nazionale Tumori-IRCCS Fondazione G. Pascale”, Naples, Italy; 2 Breast Surgery Division, “Istituto Nazionale Tumori-IRCCS-Fondazione G. Pascale”, Naples, Italy; 3 Division of Radiology - Presidio Ospedaliero di Marcianise, Caserta, Italy; 4 Pathology Division, “Istituto Nazionale Tumori-IRCCS-Fondazione G. Pascale”, Naples, Italy; 5 Medical Oncology Division, Igea SpA, Naples, Italy

**Keywords:** Breast neoplasms, Magnetics, Margins of excision, Preoperative (procedure), Reoperation

## Abstract

**Background:**

We retrospectively evaluated safety and performance of magnetic seed localisation of nonpalpable breast lesions.

**Methods:**

We reviewed records of patients with nonpalpable breast lesions preoperative localised by placing magnetic Magseed® marker between February 2019 and December 2020. During surgery, Sentimag® magnetic probe was used to localise the marker and guide surgery. Safety, lesion identification and excision with tumour with free margins and re-excision rate were assessed.

**Results:**

A total of 77 Magseed® devices were placed into the breasts of 73 patients, 44 under ultrasound and 33 under stereotactic guidance (4 bilateral). All devices were retrieved as were the target lesions. Magnetic marker placement was successful in all cases without any adverse event. Intraoperative identification and excision of the localised lesion were successful in 77 of 77 of cases (100%). In three cases (all of them calcifications with the seed placed under stereotactic guidance), the seed did not reach the exact target position of the biopsy clip; thus, larger excision was needed, with localisation failure attributed to incorrect clip insertion (*n* = 1) or to clip dislocation (*n* = 2). Migration of the marker was negligible in all patients. Complete excision after the initial procedure with at least 1-mm disease-free margins was obtained in 74 out of 77 (96.1%) lesions. The re-excision rate was 3 out of 77 (4%).

**Conclusions:**

Magnetic marker localisation for nonpalpable breast lesions was safe, reliable, and effective in terms of lesion identification, excision with tumour-free margins and re-excision rate.

## Key points

- Preoperative magnetic marker placement was successful without any adverse event.
- Preoperative magnetic marker localisation for nonpalpable breast cancer is feasible and safe.
- Preoperative magnetic marker localisation was effective in terms of lesion identification, tumour-free margins and re-excision rate.

## Background

The number of patients with nonpalpable breast lesions has increased due to the widespread improvement and use of screening mammography. The advent of vacuum-assisted tissue sampling has led to a dramatic reduction in the number of patients requiring open diagnostic surgical excision for indeterminate radiological abnormalities [1]. However, in addition to patients with nonpalpable breast cancer at initial diagnosis or after neoadjuvant therapy (NAT), open surgical excision is still required in a non-negligible number of patients for accurate diagnosis [2]. Therefore, accurate preoperative localisation of these lesions is essential to guide precise surgical excision.

Among preoperative localisation techniques, traditional wire-guided localisation (WGL) has been the most common method to localise the tumour before surgery. However, this procedure has drawbacks such as logistical challenges, migration issues (migration occur in up to 3% of patients), patient discomfort, and the risks of wire fracture while transferring the patient from the radiology department to the operating room [3]. In order to minimise patients’ discomfort and the risk of dislocation, wire localisation is usually performed on the day of surgery. The localisation wire can cause manifold complications, such as diathermy burns, pericardial injury, and wire dislocation/ transection [4]. Despite its limitations, WGL remains the standard method of localisation in most breast units, given the long-term data supporting its effectiveness.

Radioactive seed localisation (RSL) is a feasible alternative to WGL [5]. In contrast, RLS is an easy, accurate and safe technique that has been rapidly gaining acceptance among radiologists and oncology surgeons [5]. The procedure involves placing a ^125^I seed with a half-life of 60 days in the breast lesion area, which will be removed during surgery. RSL has been associated with a lower rate of positive surgical margins, as well as a lower rate of local recurrence and re-excision [5]. Furthermore, recent evidence suggests that RSL would be more cost-effective than WGL [6] and would improve the oncological outcomes of image-guided surgery [7]. However, handling of radioactive material requires special licensing and is associated with strict regulatory requirements and high administrative burden.

We report herein the feasibility, safety and performance finding of a different technology that aims to overcome disadvantages of WGL and RLS by using a magnetic marker and a magnetic detection probe. Magnetic seed, also known as Magseed® needle with magnetic marker system, was first approved by Health Canada in April 2014 and used to localise nonpalpable breast lesions [8–10]. It consists of a magnetic marker, 5 mm in length and 2 mm in diameter, which can be detected using the Sentimag® probe during surgery [8]. Sentimag® probe generates an alternating magnetic field to transiently magnetise the iron-containing seed, and this signal is then detected by the probe. The magnetic seeds can be inserted up to 30 days prior to surgery using a needle, under either ultrasound or stereotactic guidance. In addition, to overcome many of the limitations of WGL and allowing flexible scheduling by decoupling surgery and radiology, this wireless option allows for marker deployment at the time of biopsy and can be very useful in patients undergoing NAT.

The primary outcome of our study was successful localisation of the lesion (removal of the index lesion) using the predetermined localisation technique. Secondary outcomes were successful and excision of the lesion and re-excision rate.

## Methods

### Ethics and patient characteristics

Our institutional ethical committee approved the procedure. Each patient signed the informed consent. The study was executed according to up-to-date Declaration of Helsinki version and to International Conference on Harmonisation of Good Clinical Practice Guidelines.

We reviewed the records of patients who underwent preoperative breast lesion localisation through concurrent placement of the magnetic Magseed® marker (Endomagnetics, Cambridge, UK) from February 2019 to December 2020. This is a small single-use metal device (seed) of 5 × 2 mm, made of surgical grade stainless steel, nonradioactive, with a low nickel content, approved for use by the US Food and Drug Administration in 2016 for breast localisation. During surgery, the Sentimag® magnetic probe was used to localise the marker and guide surgery.

The exclusion criteria for Magseed® location were the presence of a pacemaker or device implanted in the chest wall and nickel allergy. Table 1 reports the patient characteristics.

**Table 1:** Characteristics of patients (*n* = 73) and breast lesions (*n* = 77) and performance of Magseed® preoperative localisation according to ultrasound or stereotactic guidance

Characteristics	Ultrasound guidance (*n* = 44)	Stereotactic guidance (*n* = 33)	*p*-value
*Age of patients*			
Median	52.5	56	0.34
Range	32−66	39−82	
*Side*			
Right	20	21	0.75
Left	24	22	
*Lesion size (mm)*			
Median value	10	10	0.45
Range	5−17	2−22	
*Histology*			
Benign (*n* = 18)	16	10	0.58
Malignant (*n* = 33)	28	23	
*Successful intraoperative identification and excision of the lesion*	44/44 (100.0%)	33/33 (100.0%)	1
*Complete excision after the initial procedure with at least 1 mm of disease-free margins*	41/44 (93.2%)	33/33 (100.0%)	0.13
*Re-excision rates*	3/44 (6.8%)	0/33 (0.0%)	0.13

The primary outcome of our study was successful localisation of the lesion (removal of the index lesion) using the predetermined localisation technique. Secondary outcomes were successful and excision of the lesion and re-excision rate. Any surgical margin < 1 mm was deemed positive, and patients underwent re-excision as appropriate.

### Localisation technique

**Fig. 1:**
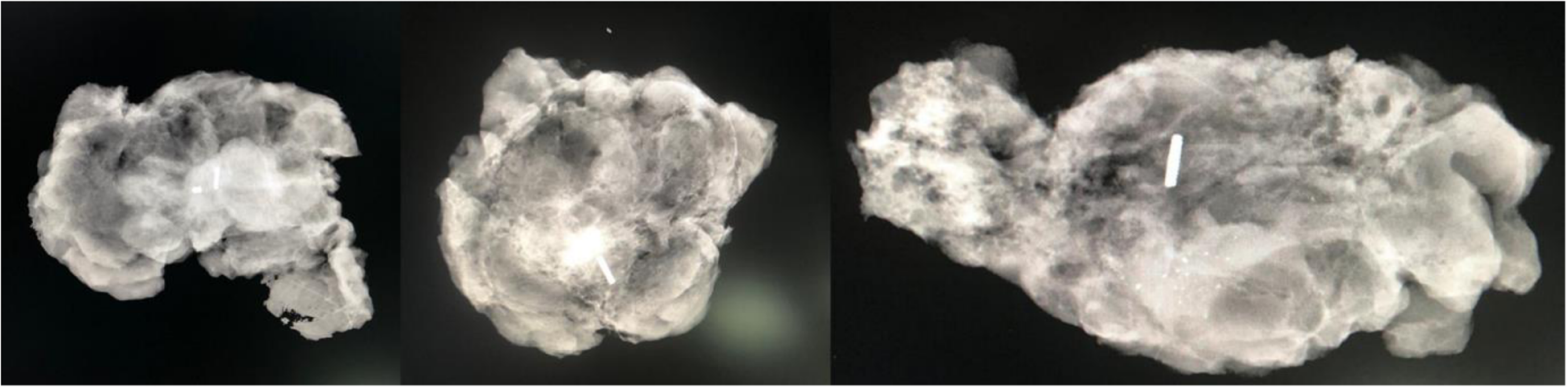
Examples of specimen radiographs showing the identification target in the centre of the lesion

Magseed® was inserted within the core of the target lesions under ultrasound guidance or mammography stereotactic guidance, a minimum of 0 day and a maximum of 14 days prior to surgery. For the two patients subjected to NAT, the Magseed® was inserted at the end of therapy on target lesion again visible. Ipsilateral two-view mammography (mediolateral oblique and craniocaudal views) was performed immediately after the procedure to confirm the seed insertion (Fig. 1).

**Fig. 2:**
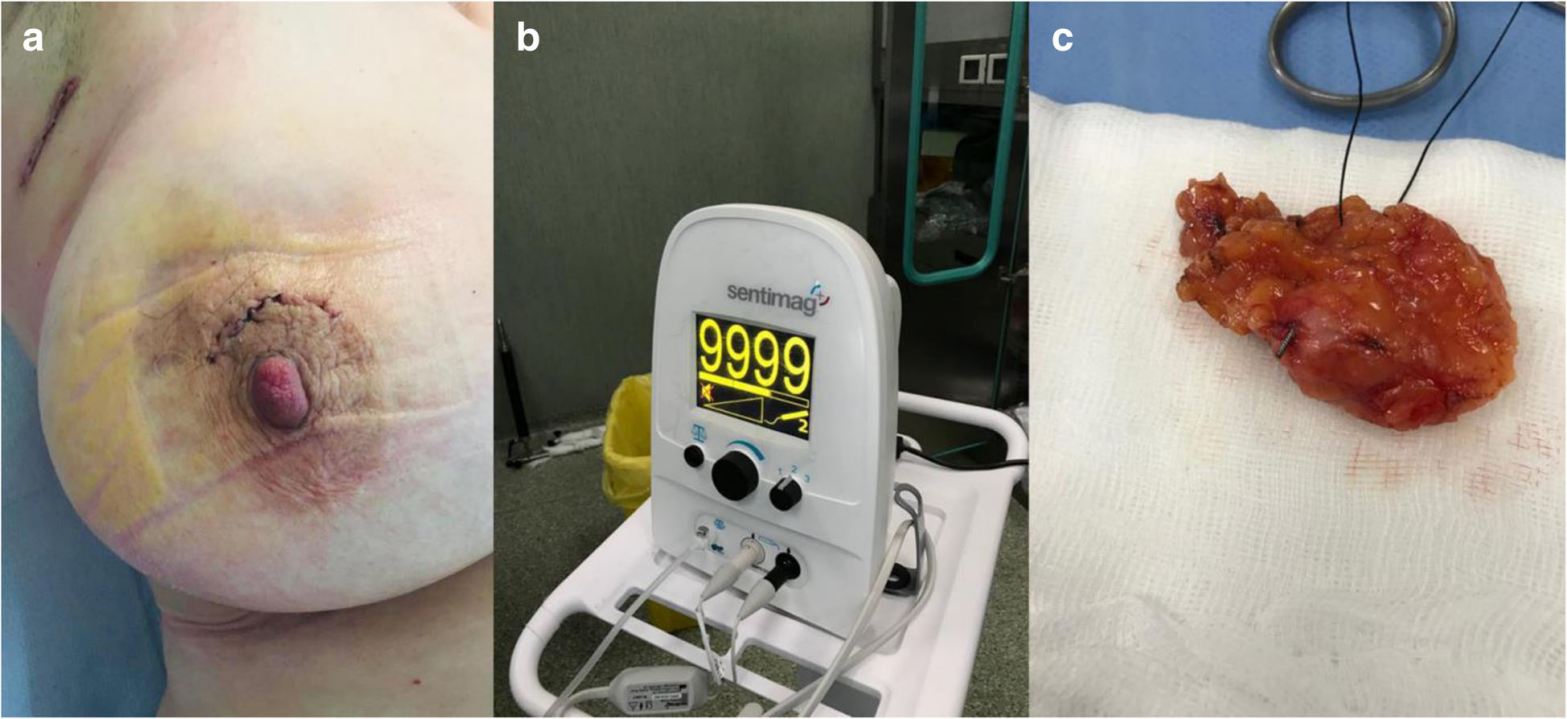
Small periareolar incision performed with preoperative lesion localisation using Magseed® and Sentimag® (**a**). Monitor showing magnetic field (**b**). Specimen containing Magseed® (**c**)

A handheld magnetometer detector, called Sentimag® probe, was used to check whether the seed was detectable in the breast. The probe generates an alternating magnetic field that transiently magnetises the iron oxide within the Magseed®. The magnetic signature from the seed is detected by the Sentimag®, and a numerical count is displayed by the probe unit along with an audio tone. Nonmagnetic surgical tool (polymer) was used to avoid interferences with the seed. The frequency varies according to the intensity of the magnetic field produced by the Magseed®, thus allowing the surgeon to gauge the distance of the seed from the probe and accurately localise the lesion (Fig. 2).

### Histopathological examination

Histopathological examination was performed on surgical specimens. Tumour was classified according to the latest 8th American Joint Committee on Cancer staging system [10]. Ductal carcinoma *in situ* (DCIS) and invasive cancers were counted as malignant lesions. All other results, including lobular carcinoma *in situ*, fibroadenoma, atypical ductal hyperplasia, and radial scar, were considered nonmalignant lesions.

### Statistical analysis

Continuous data were expressed as median value and range. Categorical data are expressed as counts and percentages. Mann-Whitney *U*-test was used to verify differences between groups of continuous variables. *χ*^2^ test was used to assess differences between percentage rates among groups. *p*-values < 0.05 were considered significant for all tests. All analyses were performed using Statistics Toolbox of MATLAB R2007a (the MathWorks Inc., Natick, MA, USA).

## Results

Seventy-three patients underwent Magseed® placement for 77 lesions, 44 under ultrasound guidance and 33 under stereotactic guidance. Four out of 73 patients had bilateral seed placed (Table 1). Median size of the lesions was 7.5 mm ranging from 2 to 22 mm. All Magseed® devices were retrieved as were the target lesions. This finding was confirmed by specimen radiography. The two lesions undergoing NAT were still visible at the end of therapy.

Magnetic marker placement was successful in all cases, and no adverse events or complications were recorded, no infections, no preoperative haematomas after seed placement, and no wound dehiscences. A single postoperative haematoma was found in a patient treated with an antiplatelet agent suspended 7 days before surgery and replaced with low-molecular-weight heparin, considered not related to the device.

Intraoperative identification of Magseed® and excision of the localised lesion were successful in 100% of lesions (77 of 77) by different breast surgeons with different years of experience (Table 1). In three cases, the seed did not reach the exact position of the target. In those three cases, seed had been placed under stereotactic guidance for the presence of calcifications, and the positioning was difficult. The marker was found placed 2 to 3 cm distant to stereotactic vacuum assisted breast biopsy clip; thus, larger excision was needed to get the target because specimen x-ray did not reveal the calcifications. Two of these cases were proven to pure DCIS at histopathological analysis, and one had undergone NAT. The localisation failure of these three cases was attributed to incorrect insertion of the clip (*n* = 1) or to the clip dislocation (*n* = 3). Migration of the magnetic marker was negligible in all patients.

Complete excision after the initial procedure with at least 1-mm disease-free margins was verified in 74 out of 77 lesions (96.1%). Final pathology report showed close margins < 1 mm; thus, all underwent margin re-excision. Reoperation rate was of 4% (3 out of 77 patient).

## Discussion

The aim of this study was to evaluate the preoperative magnetic seed (Magseed®) placement and intraoperative localisation by Sentimag® probe in nonpalpable breast lesions in terms of feasibility, safety and performance. We demonstrated a high successful localisation and retrieval rate of 100%, positive margins < 1 mm only in three patients and a very low re-excision rate (4%). No migration of the marker was observed. In only three cases, the seed did not reach the exact position of the target; all of them performed under stereotactic guidance, due to clip dislocation (*n* = 2) or incorrect insertion (*n* = 1). These findings support the effective utilisation of the Magseed® magnetic marker and the Sentimag® probe as a valid and safe alternative to WGL and RSL and confirm the results of previous studies.

Harvey et al. [5] reported no magnetic marker migration comparing mammograms performed after Magseed placement, 29 of 29 Magseed® being within 10 mm, with 27 of 29 seed placed at the target lesion and the other two seeds at 2 mm and 3 mm from the target. The magnetic seeds were placed with a median of 5 days prior to surgery (range 1 to 15 days), and no complications or adverse events were observed due to magnetic seed placement of the surgery. Lamb et al. [11] reported a technical success (*i.e*., the placement of magnetic seed within 1 cm from the target) for 206 of 213 markers (96.7%), 7 (3.3%) being beyond 1 cm from the target. All magnetic seeds (213 of 213) were removed successfully at surgery, and no complications were observed. In the study by Pohlodek et al. [12], localisation and detection of all magnetic seeds were reported, and neither migration nor complications were observed. Price et al. [13] reported a successful procedure for localisation of 73 out of 73 (100%) nonpalpable breast lesions within 1 cm from the target, but only 70% of all magnetic seeds were within 1 mm from the target. All magnetic seeds were successfully retrieved, and three complications (4%) were reported including postoperative haematoma, postoperative infection and pneumothorax, which was likely due to difficulty in finding the magnetic seed. One small study [14] had all magnetic seeds localised within a median of submillimetre distance from the target and successful marker identification in 15 out of 15 cases. The study also assessed surgeon’s satisfaction with the magnetic seed technology, and all surgeons were strongly satisfied and reported that they are able to adopt this new technology.

Several advantages of the use of magnetic seed for preoperative localisation of breast lesions can be highlighted. First, this magnetic technique is radiation-free and spares both patients and clinicians from radiation exposure. Second, the possibility to insert the seed several days before surgery (a single appointment localisation in daily workflow) helps to schedule operating theatre avoiding waste of time and delayed breast surgery, whilst wires suffered from the risk of dislocation. In fact, since February 2020, Magseed® has received an extended indication in Europe for long-term use before neoadjuvant chemotherapy; so far, they can be placed several months before surgery. Third, the time needed per procedure could be relatively short, at least if compared with that needed for RSL. A small non-randomised study done in Germany found that time spent in preoperative care using the Magseed®-Sentimag® system (5.4 ± 1.3 min, mean ± standard deviation) was significantly lower than for the ^99^Tc-based RSL group (82 ± 20 min) (*p* < 0.0001) [14]. The authors mentioned that being the system nonradioactive, no need for special precautions are needed [14, 15]. Lastly, cosmetic outcome is well improved by small incisions that are commonly allowed by the magnetic seed technique, thus increasing patient satisfaction and quality of life. At our institution, both radiologists and surgeons confirmed to feel safe and comfortable with the magnetic seed technique and highlighted the fast learning curve they needed to perform magnetic-guided insertion and surgery.

An important aspect of the Magseed® preoperative localisation is a signal void artefact on magnetic resonance images [16, 17], crucial in assessing response to NAT, considering that contrast-enhanced magnetic resonance imaging is the best recommended imaging method in this setting to assess therapy response [17–23]. Depending on the MRI sequence, the Magseed can create a bloom artefact which measures up to 4 cm. This may hamper the detection of residual disease after neoadjuvant systemic therapy if MRI is required for monitoring response to treatment. Nevertheless, contrast-enhanced mammography is an effective alternative imaging modality which may be considered if signal void artefacts pose a significant issue [18, 19].

Finally, the cost of the procedure should be considered. The cost of the Sentimag®-Magseed® system was estimated in the UK at £226 per procedure, while the cost of standard care using isotope and blue dye in combination is £194 per procedure [16]. So, while standard WGL is certainly cheaper than magnetic seed localisation, the cost of this new approach could be lower than that of RLS.

Limitations of our study are the small size of the sample, the monocentric nature, of the study and the absence of a control group. Anyway, we add some more evidence to the safety and good performance of magnetic markers for preoperative breast lesion localisation.

In conclusion, our study confirmed the positive experience with the magnetic preoperative localisation of nonpalpable breast lesions. This approach showed to be safe, reliable and effective in terms of lesion identification, excision with tumour-free margins and re-excision rate.

## Abbreviations

DCIS: Ductal carcinoma *in situ*
NAT: Neoadjuvant therapy
RSL: Radioactive seed localisation
WGL: Wire-guided localisation
